# Design principles for molecular animation

**DOI:** 10.3389/fbinf.2024.1353807

**Published:** 2024-08-21

**Authors:** Stuart G. Jantzen, Gaël McGill, Jodie Jenkinson

**Affiliations:** ^1^ Science Visualization Lab, Biomedical Communications, Department of Biology, University of Toronto Mississauga, Mississauga, ON, Canada; ^2^ Biocinematics, Victoria, BC, Canada; ^3^ Center for Molecular and Cellular Dynamics, Department of Biological Chemistry and Molecular Pharmacology, Harvard Medical School, Boston, MA, United States; ^4^ Digizyme, Brookline, MA, United States

**Keywords:** molecular visualization, science animation, dynamic visualization, molecular motion, science communication, visual complexity, design principles, pedagogy

## Abstract

Molecular visualization is a powerful way to represent the complex structure of molecules and their higher order assemblies, as well as the dynamics of their interactions. Although conventions for depicting static molecular structures and complexes are now well established and guide the viewer’s attention to specific aspects of structure and function, little attention and design classification has been devoted to how molecular motion is depicted. As we continue to probe and discover how molecules move - including their internal flexibility, conformational changes and dynamic associations with binding partners and environments - we are faced with difficult design challenges that are relevant to molecular visualizations both for the scientific community and students of cell and molecular biology. To facilitate these design decisions, we have identified twelve molecular animation design principles that are important to consider when creating molecular animations. Many of these principles pertain to misconceptions that students have primarily regarding the agency of molecules, while others are derived from visual treatments frequently observed in molecular animations that may promote misconceptions. For each principle, we have created a pair of molecular animations that exemplify the principle by depicting the same content in the presence and absence of that design approach. Although not intended to be prescriptive, we hope this set of design principles can be used by the scientific, education, and scientific visualization communities to facilitate and improve the pedagogical effectiveness of molecular animation.

## 1 Introduction

The design of molecular representations is based on the same underlying graphical principles that drive other fields. Regardless of subject matter, we typically develop and use conventions that help guide the viewer’s attention to features of the display that support our main communication objectives. We colorize, explore alternate framings and sometimes even selectively delete, fade or blur to emphasize the elements most relevant to the message or story. Molecular visualization is now a mature field where issues of representation, color and their myriad combinations have been mapped to specific communication goals/intentions and, in this way, a certain degree of codification has been useful. Various structural representations abstract away from atomic-level features toward higher order concepts. In contrast to space-filling or ball-and-stick models that depict molecules in atomic detail, ribbon diagrams of proteins originally designed by Jane Richardson have transformed our understanding of secondary structure and their many combinations into higher order structural domains by focusing the viewer’s attention on the protein backbone (Richardson, 1981). Similarly color – whether applied to individual atoms as in the Corey–Pauling–Koltun (CPK) convention, or the red and blue colors used to depict electrostatic surface potentials – has been codified to capture properties of the constituent parts and regions of molecules. As well, more recent efforts have been focused on establishing best practices in molecular visualization (Garrison and Bruckner, 2022). All of these standard characteristics and practices have in large part been focused on static representations of molecules.

Molecules, however, are highly dynamic entities and their function is predicated on their ability to move within complex environments and also to alter their conformations in function of that environment and a multitude of interaction partners. Although some visual conventions have been explored to depict molecular motion, such as onion skinning in programs like MoFlow (Dabdoub et al., 2015), overlaying multiple structural states with partial opacity or appending arrows to represent force vectors onto moving parts of a molecule (Jenkinson, 2017), these were primarily developed to represent molecular motion in static media.

## 2 Challenges associated with dynamic representation of molecular scale phenomena

The challenges of representing molecular entities dynamically are manifold and include consideration of spatial and temporal scale, translation of unintuitive and complex overlapping motions into a decipherable visual language, and perhaps most importantly attention to the needs of the audience for whom these visualizations are designed. Molecular biology is replete with phenomena that provide great challenges for visualization and understanding. Spatial and temporal scales are hard to comprehend and span several orders of magnitude. A water molecule is about 0.2 nm in size while DNA molecules can be centimeters long. The atoms in a cytoplasmic protein vibrate at ∼1,012 times per second, yet the molecule might take several seconds to diffuse across a cell.

Just as the spatial and temporal scales of molecular environments are challenging to convey, the qualities of motions and interactions are equally complex. It’s easy to imagine long linear molecules like DNA as being flexible; it’s perhaps harder to appreciate that all molecules have varying degrees of internal motion, where atoms, chemical groups, and domains explore the thermodynamic energy landscape of conformational change. This leads to nuanced intermolecular interactions as well, where orientations, positions, and conformations must be permissive for successful and stable binding to occur. Finally, because molecules are smaller than the wavelength of light, our common visual experience of colored, reflective surfaces ceases to apply.

### 2.1 Target audience considerations

Careful decisions must be made about what is presented and how it is represented when visually depicting a molecular process. Which molecules are shown, and how many of each? How fast do molecules move and what is the nature of their motion? Much of the decision-making vis-à-vis representation of these features is dependent upon the communication goals and the intended target audience. In peer-to-peer communication within the scientific research community, a high level of abstraction is acceptable, particularly when communicating increasingly complex data (Johnson and Hertig, 2014). Although visualization of molecular simulation trajectories has been a powerful way for researchers to inspect dynamic data (Kozlıkova et al., 2017; Hildebrand et al., 2019), animation can also serve as a useful tool within the scientific community for guiding and refining research hypotheses (Iwasa, 2015), as well as an opportunity for data integration and knowledge synthesis (McGill, 2022). It is also worth noting however, that the perceived accuracy and usefulness of more delineative molecular animations is influenced by whether experts take them seriously as a medium with which to credibly communicate complex phenomena (Iwasa, 2015; Jantzen et al., 2015).

Beyond sharing research, biologists and educators are also tasked with communicating to more general audiences who lack the requisite background knowledge and subject matter expertise to directly interpret biomolecular data. In the context of undergraduate biology education, a number of frameworks provide specific learning objectives, many of which relate to students’ understanding of molecular structure and function. In particular, the BioCore (Brownell et al., 2014) and BioSkills (Clemmons et al., 2020) guides delineate learning goals that help to articulate the core concepts and competencies outlined in the Vision & Change report (AAAS, 2011; Branchaw et al., 2020). More recently, a set of nationally endorsed granular learning objectives that build upon the existing frameworks have also been proposed (Hennessey and Freeman, 2023). Other frameworks, like BioMolViz (Shor et al., 2021), cater specifically to instructors of biomolecular structure and dynamics. Taken together, these educational resources not only provide context for the relevance of the concepts addressed by the 12 Principles of Molecular Animation, but also help guide specific design decisions. Indeed, communicating the complexity and chaotic richness of the molecular world to students represents a significant challenge, and which information is included or abstracted away in a molecular animation primarily comes down to the learning objectives behind a communication piece.

Understandably, different visual solutions are required to communicate to novice learners. But what is the correct degree of simplification? One might assume that the best visualizations present an unadapted translation of a process, showing events exactly as they might proceed inside a cell. However, this information can be too much, too fast, and too complex, to directly visualize. In order to communicate effectively, the designer of educational visualizations must necessarily simplify, that is, use approximations and interpretive representations and abstractions since the molecular reality is too complex and ephemeral. However, as noted by Johnson and Hertig (2014), representations that are familiar to molecular biologists are only abstract shapes to most other audiences, who often assume incorrectly that such representations have some physical reality. In other words, the more novice the audience, the less one may be able to afford abstraction. When we consistently avoid complexity for the sake of clarity, misconceptions may remain in place or, even worse, may be established or reinforced.

### 2.2 Visualization and misconceptions

Common misconceptions held by science students relate to the belief that molecules have agency or purpose, which manifests as ligands aiming for receptors and enzymes pursuing or vacuuming up substrate (Tibell and Rundgren, 2010; Jenkinson and McGill, 2012; Jenkinson and McGill, 2013). While most students understand molecular diffusion and Brownian motion in general terms (particularly in an ideal gas scenario), they have a hard time extending those principles to real biological events. In truth, these misconceptions persist among even more proficient learners (upper level undergraduates) and their confidence in these erroneous beliefs increases over time (Gauthier et al., 2019). These misconceptions may be compounded by the way in which molecular scale phenomena are represented.

If, for some subset of educational animations, we prioritize depicting dynamic molecular “realism,” might this influence how students conceptualize intracellular environments and events? Certainly, by devoting attention to fundamental molecular behaviors when designing visuals we may allow for a more robust understanding of the spaces in which these events take place. This is a question that, to-date, remains unanswered, but represents a burgeoning area of inquiry (Cooper et al., 2015; Jenkinson, 2018; Procko et al., 2022). Certainly, an important early step is to identify which concepts could best reflect the dynamic realism of these environments and to create example visuals to help guide animation practitioners in the development of molecular visualizations.

## 3 Representing the emergent properties of molecular environments

In representing dynamic environments, animators strive to capture the forces of the natural world that act upon entities. In so doing, they allow the viewer to suspend disbelief; that is, to believe in the illusion of movement.

### 3.1 The twelve principles of animation

The “12 principles of molecular animation” (described in detail below) are partly inspired by, but also exist in contrast to a more famous set of principles, introduced by Disney animators Frank Thomas and Ollie Johnston in their book “The Illusion of Life” (1981). More recently, this list of concepts was adapted for computer animation by Lasseter (1998), the former CEO of Pixar Animation Studios. These principles are: squash and stretch, anticipation, staging, straight ahead action versus pose to pose, follow through and overlapping action, slow in and slow out, arcs, secondary action, timing, exaggeration, solid drawing, and appeal. Solid drawing does not appear in the computer animation paper, presumably because in this newer medium, visuals are generated in three-dimensional space, greatly alleviating the need for animators to predict how a volume moves through space.

The goals behind these traditional animation principles are multifactorial. They are intended to create more convincing and engaging animations, ensuring that on-screen characters have weight, volume, and inertia, and that the audience can clearly follow actions and events. In contrast, constraining molecular movements to macroscopic physics is not only factually incorrect but, more importantly, misleading to novice audiences. Principles like “slow in/slow out” and “follow through” may make computer-generated movements feel natural, however real molecular motion is far more chaotic. The principle of “arcs” reminds animators that structures such as appendages and projectiles rarely travel in straight lines. It is true that molecules don’t move in straight lines, but neither do they travel in arcs. By applying character animation principles to molecules, are they given anthropomorphic or zoomorphic properties?

The twelve principles of animation, as outlined by Thomas, Johnston, and Lasseter, should not be discarded en masse. They are very useful tools for animators and in many cases separate amateur animation from higher quality pieces. Several of these animation principles aid in guiding the audience, as discussed below. However, the molecular animator should be aware of when and how these principles can be beneficial and when they may obscure the true nature of molecular behaviors. Perhaps the inherent conflict between abstraction and realism is this: molecules do not behave the way we intuitively expect (or at least a novice learner expects), therefore depictions that make use of traditional animation principles belie reality and promote misconceptions. On the other hand, whereas realistic depictions are complex and challenging to follow, is there a balance to incorporating these opposing approaches in molecular animation?

### 3.2 Twelve principles of molecular animation

Inspired by Disney’s twelve principles of animation, we have identified twelve concepts (Table 1) that we believe are important to consider when creating *molecular* animations. Many of the concepts pertain to misconceptions that have been identified in the literature relating to biology education, while others are derived from an environmental scan of commonly misrepresented features of molecular environments (particularly in relation to core concepts in biology education). Many core concepts associated with cell biology have been identified and codified in so-called concept inventories (Ellis, 2001; Garvin-Doxas and Klymkowsky, 2008; Robic, 2010; Newman et al., 2017). Subject areas of particular difficulty for students include protein conformational change and stability (Robic, 2010), diffusion and random molecular motion (Garvin-Doxas and Klymkowsky, 2008; Jenkinson and McGill, 2012; Gauthier et al., 2019), and molecular crowding (Ellis, 2001; Gauthier et al., 2019). Learners have tremendous difficulty coming to terms with the full complexity of the molecular world, given the perceived efficiency of biological systems. Through the design of these principles, we have proposed what we believe to be important considerations for the representation of these concepts.

**TABLE 1:** Twelve principles for molecular animation.

Principle number	Title	Description	Link
1 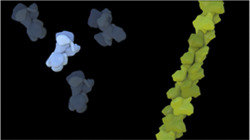	Molecules move through random collisions	Molecules move around through collisions resulting in random brownian motion.	Treatment A: https://vimeo.com/826294674 Treatment B: https://vimeo.com/826294708
2 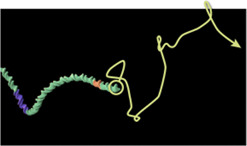	Long molecules experience similar forces along their length	The same forces are present along the full length of a long molecule. Putting a head to a molecule invokes agency.	Treatment A: https://vimeo.com/826294819 Treatment B: https://vimeo.com/826294848
3 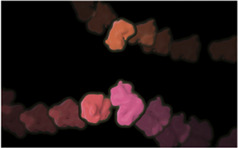	Molecules are in constant motion	Newton’s first law states that objects remain in motion without external forces. While molecules are subjected to constant forces from all sides, the result is they are in constant motion and do not start and stop spontaneously.	Treatment A: https://vimeo.com/826294884 Treatment B: https://vimeo.com/826294915
4 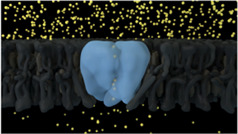	Intermolecular attractions are local forces	At this scale, showing negative pressure or distant molecules flooding toward a target invokes agency. The same applies to the relative motion between two binding partners.	Treatment A: https://vimeo.com/826294760 Treatment B: https://vimeo.com/826294787
5 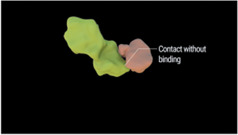	Unproductive collisions occur more often than productive ones	Molecules are in constant collision. However, encounters between complementary molecules do not necessarily result in binding. Statistically, there are likely to be many more unproductive collisions than productive ones.	Treatment A: https://vimeo.com/826294723 Treatment B: https://vimeo.com/826294744
6 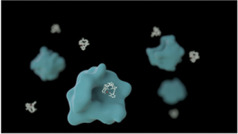	Many instances of molecules and events exist	There are typically many instances of molecules and events present in a given environment; repetition can also reinforce the process being depicted.	Treatment A: https://vimeo.com/826295482 Treatment B: https://vimeo.com/826295519
7 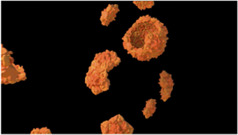	Not all instances of a molecule change state in a process	Not every molecule is used in a process or changes its state. More monomers are present than will be incorporated into a polymer, and typically more substrates are present than will be converted into a product. Likewise, not all molecules will cross a barrier or will bind to a chelator.	Treatment A: https://vimeo.com/826294564 Treatment B: https://vimeo.com/826294638
8 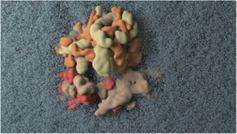	Light-based effects are not observed at a molecular scale	Careful consideration must be given to the visual effects that are employed and the messages they convey. For example, water is composed of molecules and light does not interact with it at the molecular scale to produce macroscopic phenomena, like caustics, refraction, distortion, or crepuscular/god rays. These “underwater” effects are not relevant at the molecular scale.	Treatment A: https://vimeo.com/826295060 Treatment B: https://vimeo.com/826295087
9 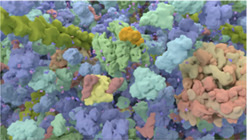	Molecular landscapes are crowded and diverse	Cellular environments are busy and crowded, with very little empty space, particularly if molecular water is included. Even without the depiction of molecular water, macromolecules take up a sizeable percentage of the volume.	Treatment A: https://vimeo.com/826295171 Treatment B: https://vimeo.com/826295211
10 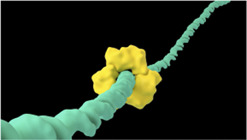	Molecules are physical entities with definable boundaries	Intersecting surface meshes provide conflicting or obscured information about interaction and binding sites.	Treatment A: https://vimeo.com/826294939 Treatment B: https://vimeo.com/826295005
11 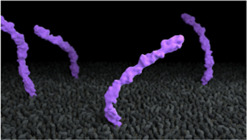	Proteins exhibit a range of flexibility	Proteins are not rigid bodies and instead exhibit a range of flexibility - at the atomic, side chain and domain levels. It is important to represent these movements since they are often closely tied to protein function.	Treatment A: https://vimeo.com/826295350 Treatment B: https://vimeo.com/826295444
12 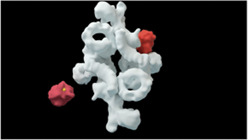	Many binding reactions are reversible before reactions occur	Collisions may result in binding events between molecules but these events are not permanent. Many reactions are reversible at the individual molecule level.	Treatment A: https://vimeo.com/826295263 Treatment B: https://vimeo.com/826295314

**FIGURE 1:**
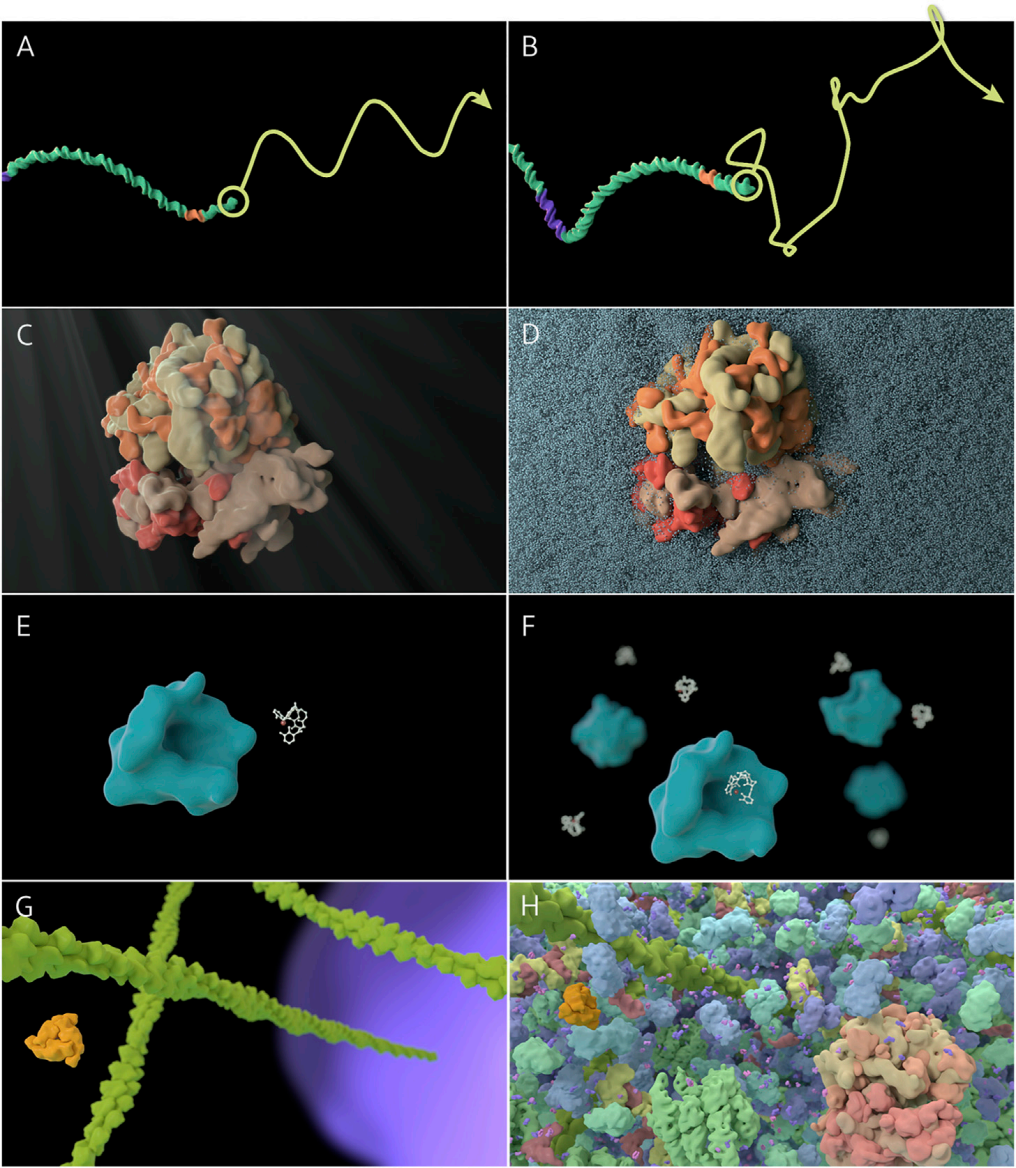
Thumbnails of sample principles (animations may be viewed at www.sciencevis.ca). **(A, B)** Principle 2: Long molecules experience similar forces along their length; Treatment I: Trajectory shows sinusoidal motion; Treatment II: Trajectory shows random motion. **(C, D)** Principle 6: Many instances of molecules and events exist; Treatment I: A single enterobactin molecule binds to a single siderocalin molecule; Treatment II: Several enterobactin molecules bind to several siderocalin molecules. **(E, F)** Light and molecular water do not produce macroscopic phenomena; Treatment I: Ribosome with caustics, ripple distortion, and crepuscular rays (“God rays”); Treatment II: Ribosome partially surrounded by water molecules. **(G, H)** Principle 9. Molecular landscapes are crowded and diverse; Treatment I: Cytoplasm environment includes actin filaments only; Treatment II: Cytoplasm includes a diverse set of proteins, tRNA, and small molecules.

Each principle is titled with a biological concept, includes a learning objective related to the concept (Table 1), and is illustrated by two short paired animations showing the principle in action (Figure 1). The paired animations use a simplified biological process to demonstrate the principle; one shows the process as it is typically depicted (without incorporating the principle), the other shows the same process with the principle present (links to animations are included in Table 1). As well, we have included in our website (https://sciencevis.ca/index.php/portfolio/molecular-visualization-principles/), actionable suggestions for how each principle might be applied. The aesthetic design of the animations was determined primarily to make the principles as clear as possible. We derived the models from structural data (PDB), but we also simplified the geometry representing molecular surfaces and applied bright, saturated colors. These last two decisions were made to demonstrate that not only highly detailed or “photorealistic” representations would warrant the incorporation of these principles. In each case, we were forced to ignore a subset of the other principles in a given animation on a case by case basis, again to ensure the principle being presented was demonstrated in the clearest way possible. As noted by Tibell and Rundgren (2010), no single visualization can convey all the critical aspects of knowledge, and so, we must be selective in choosing what to include in order to convey specific learning objectives. In a formal learning environment this may require using multiple representations.

We previously argued for organizing, annotating, and presenting the data sources used in creating scientific animations (Jantzen et al., 2015). The properties of elements to which data sources can be linked are structure, appearance, motion, interactions, and populations. In other words, the resources used to inform structure are often distinct from those used for motion, for example, Our principles are most closely related to motions, interactions, and populations. Developing useful resources and workflows for incorporating these kinds of data and concepts is a task both for biologists and molecular animators. To-date, data-driven structural representations are far more prevalent in molecular animation than data-driven representations of molecular motion or interactions. Although the visualization of molecular trajectories issued from simulation methods (like molecular dynamics or other coarse-grained approaches) are common in the scientific research community, these simulation trajectories are seldom used in the context of educational molecular animations.

The molecular animation principles described here are not intended to be prescriptive, but rather are presented to raise awareness for the biophysical properties of molecules, and to encourage the careful consideration of how novice audiences might respond to molecules being shown with more or less agency. When science animators are creating molecular animations, they have many choices to make, and it is our hope that these content creators will carefully examine their learning objectives and make rational choices based on these.

## 4 Discussion

Attempting to show a molecular environment in all its realistic complexity is not possible, and even a sophisticated approximation can be very difficult for viewers to extract pertinent information from. Hence our attempt to deconstruct some of the complexity of molecular representation into a number of concepts that can be brought into animations largely independently from each other, as deemed appropriate. Nevertheless, the principles we have listed almost all add information and possibly cognitive load to the viewing experience, thereby potentially affecting learning. Although further studies will be needed to measure exactly how the implementation of these principles may positively or negatively impact students’ learning gains, we are encouraged by the fact that these concepts map well to specific learning objectives from many common educational frameworks. In reference to the BioCore guide, our principles 1 through 7 relate to “Transformations of Energy & Matter” and specifically support the idea that a molecule moves via random motion and its “movement is affected by its thermal energy, size, electrochemical gradient, and biochemical properties.” Principles 7 through 12 relate to “Structure and Function” and, in particular, support the concept that “the three dimensional structure of a molecule and its subcellular localization impact its function, including the ability to catalyze reactions or interact with other molecules” (Brownell et al., 2014). In the context of the BioMolViz framework, our principles are not only relevant to the “Molecular interactions,” “Structural Model Skepticism” and “Molecular Dynamics” portion of the framework, but specifically support difficult concepts like evaluating the flexibility and disordered regions of a macromolecule (i.e., principles 2 and 11) (Shor et al., 2021). More generally, our molecular animation principles have the potential to raise awareness among instructors and, by extension, among students about the importance of modeling, information literacy and assessing the credibility of information presented in scientific communications - all key competencies promoted in Vision and Change and the Bioskills guide (Clemmons et al., 2020).

Although the 12 principles address important aspects of molecular depiction, our intent is not to be prescriptive but rather to remind visualization practitioners of these concepts while taking into consideration the myriad variables involved in designing a molecular animation for a specific audience. Fortunately, there are many ways in which a designer can promote clarity while incorporating molecular animation principles. Werner (2022) provides a thoughtful discussion on the strategies to consider in the production of molecular animation. These include approaches to cinematic storytelling, considerations in the representation of molecular structures, and strategies for differentiating between various levels of molecular motion. Additionally, one can take advantage of established principles of design, leveraging preattentive features to guide the viewer’s attention, such as color, contrast, and motion. Accessory elements such as arrows, glows, and labels are also indispensable for guiding attention and helping viewers fully understand the narrative. One can also reduce the depth of field in a three-dimensional view to focus attention, or assign less saturated colors to less important elements. The virtual camera used to frame scenes is a powerful tool for guiding a narrative. One can use it to show only what audiences should attend to, thus reducing split attention effects. Because it is harder to immediately grasp the scale and organization of a molecular landscape, it is important to maintain context and continuity – both temporally and spatially. Variation in temporal scale may be communicated in a number of ways, including sonification cues, manipulation of the timeline, or perhaps most accurately including a timescale to remind viewers of the rate at which these activities occur (Le Muzic et al., 2015; Eschner et al., 2023). In maintaining spatial context, panning or tracking allows the camera to follow a character, while zooming in and out focuses attention and shows greater context, respectively. One might also employ a transition between more biophysically accurate representations and more simplified representations in order to convey spatial scale while maintaining complexity. However, care must be taken that the audience understands the transition for what it is, and does not assign biological meaning to it. Ultimately, as McGill (2014) reminds us, a representation of molecular-scale phenomenon is just that – an interpretation of scientific data that is not intended to be synonymous with the science informing its design.

### 4.1 Next steps

With the twelve principles presented here, we have merely scratched the surface of biophysical concepts that can be visualized. There are certainly other molecular principles for which different representational styles are worth considering. There are also phenomena at larger or smaller scales that are worth careful consideration, for example, the physical quality of lipid membranes at the cellular scale or the oscillations of atoms involved in covalent bonding. We eagerly anticipate dialog on whether our format of pairs of animations used to illustrate these concepts is a useful one.

There is still much to be learned about the role of animation in correcting misconceptions and deepening understanding of molecular biology. Studies undertaken to-date are promising, but there is still much to uncover before general recommendations and statements can be made that will effectively guide the design of dynamic molecular representations. Educational studies measuring the pedagogical effectiveness of molecular animations that incorporate these principles could help validate the importance of such design decisions when trying to address specific learning objectives.

## Data Availability

The original contributions presented in the study are included in the article/supplementary material, further inquiries can be directed to the corresponding author.
